# Supplementary material for: Asynchronous magnetic resonance elastography: Shear wave speed reconstruction using noise correlation of incoherent waves
Source: Magn Reson Med. 2022 Oct 27;89(3):990–1001. doi: 10.1002/mrm.29502 (PMC9792433; doi:10.1002/mrm.29502)
Supplement: Supplementary file 1 — DATA S1 MATLAB code used to generate the numerical simulations of Figure 1 [file MRM-89-990-s002.zip › k-Wave/helpfiles/makeSphere.html]

makeSphere :: Functions (k-Wave)


# makeSphere

Create a binary map of a sphere within a 3D grid.

## Syntax

```
sphere = makeSphere(Nx, Ny, Nz, radius)
sphere = makeSphere(Nx, Ny, Nz, radius, plot_sphere)
sphere = makeSphere(Nx, Ny, Nz, radius, plot_sphere, binary)
sphere = makeSphere(Nx, Ny, Nz, radius, [], binary)
```

## Description

`makeSphere` creates a binary map of a spherical shell (using an extension of the midpoint circle algorithm) within a three-dimensional grid. The sphere position is denoted by 1's in the matrix with 0's elsewhere. If the Boolean input parameter `binary` is set to `false` (the default), the sphere map is returned as a double precision matrix. If it is set to `true`, the map is returned as a logical matrix. A visualisation of the 3D grid returned by calling `makeSphere(64, 64, 64, 20, true);` and a cut away showing the singel grid point thickness (for illustrative purposes only) are given below.


## Inputs

|  |  |
| --- | --- |
| `Nx, Ny, Nz` | size of the 3D grid [grid points] |
| `radius` | sphere radius [grid points] |

## Optional Inputs

|  |  |
| --- | --- |
| `plot_sphere` | Boolean controlling whether the sphere is plotted using `voxelPlot` (default = `false`) |
| `binary` | Boolean controlling whether the sphere map is returned as a double precision matrix (`false`) or a logical matrix (`true`) (default = `false`) |

## Outputs

|  |  |
| --- | --- |
| `sphere` | 3D binary map of a sphere |

## Examples

- Focussed Detector in 3D
- 3D Time Reversal For A Spherical Sensor

## See Also

`makeBall`, `makeCartSphere`, `makeCircle`, `makeSphericalSection`
